# Supplementary material for: Diagnostic performance of serum interferon gamma, matrix metalloproteinases, and periostin measurements for pulmonary tuberculosis in Japanese patients with pneumonia
Source: PLoS One. 2020 Jan 9;15(1):e0227636. doi: 10.1371/journal.pone.0227636 (PMC6952104; doi:10.1371/journal.pone.0227636)
Supplement: S1 Table — (DOC) [file pone.0227636.s001.doc]

**SUPPLEMENTARY INFORMATION (Table S1)**

**Table S1-**Causative bacteria in patients with non-tuberculous pneumonia

|  |  |
| --- | --- |
|  |  |
|  |  |
|  |  |
|  |  |
|  |  |
|  |  |
|  |  |
|  |  |

| *Haemophilus influenzae* |  |
| --- | --- |
| *Streptococcus pneumoniae* |  |
|  |  |
|  |  |
| *Legionella pneumophila* |  |
| *Moraxella catarrhalis* |  |
| *Haemophilus parahaemolyticus* |  |
| *Staphylococcus aureus* |  |
| Unknown |  |

Data are shown as percentage (number).

|  |  |  |
| --- | --- | --- |
|  |  |  |
|  |  |  |
