# Supplementary material for: Diagnostic performance of serum interferon gamma, matrix metalloproteinases, and periostin measurements for pulmonary tuberculosis in Japanese patients with pneumonia
Source: PLoS One. 2020 Jan 9;15(1):e0227636. doi: 10.1371/journal.pone.0227636 (PMC6952104; doi:10.1371/journal.pone.0227636)
Supplement: S2 Table — (DOC) [file pone.0227636.s002.doc]

**SUPPLEMENTARY INFORMATION (Table S2)**

|  |  |
| --- | --- |
|  |  |
|  |  |
|  |  |
|  |  |
|  |  |
|  |  |
|  |  |
|  |  |

**Table S2-**Factors associated with elevated MMP-1 levels by multivariate analysis

| MMP-1 | β coefficient | p-value |
| --- | --- | --- |
| TB | 0.004 | 0.34 |
| Malignancy | 0.59 | -0.063 |
