# Supplementary figures and images for: Diagnostic performance of serum interferon gamma, matrix metalloproteinases, and periostin measurements for pulmonary tuberculosis in Japanese patients with pneumonia
Source: PLoS One. 2020 Jan 9;15(1):e0227636. doi: 10.1371/journal.pone.0227636 (PMC6952104; doi:10.1371/journal.pone.0227636)

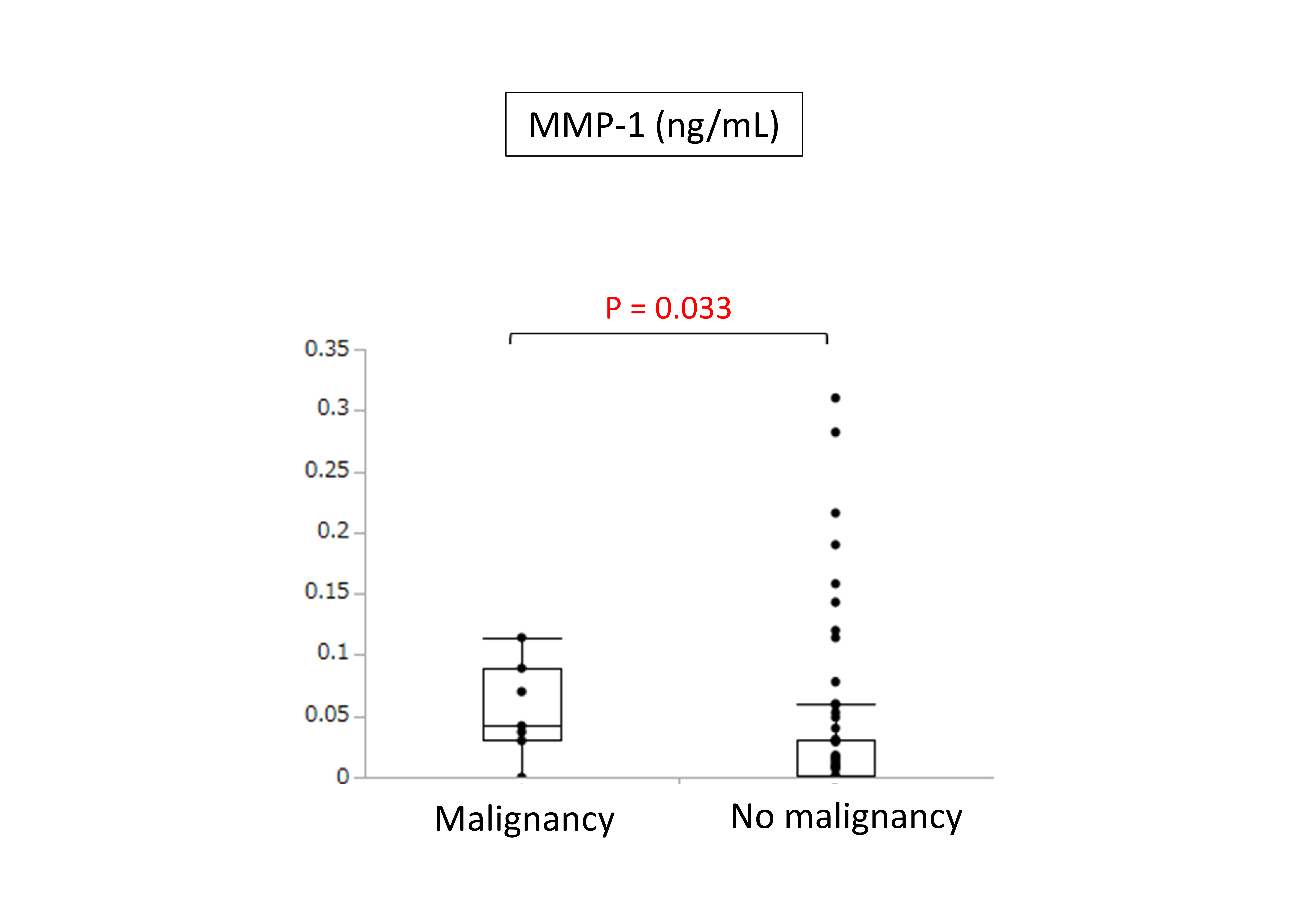

Supplement: S1 Fig — (TIFF) [file pone.0227636.s003.tiff]
